# Phenotypic evolution of appearance quality and cooking and taste quality of hybrid rice over the past 40 years in China

**DOI:** 10.3389/fpls.2024.1512760

**Published:** 2024-12-24

**Authors:** Jiongjiong Fan, Wei Li, Ying Bian, Zhengjiu Zhang, Ruoju Yang, Xia Xu, Benyi Cheng, Shihua Yang, Jianli Wu, Xiaobo Zhang, Junyi Gong

**Affiliations:** ^1^ State Key Laboratory of Rice Biology and Breeding, China National Rice Research Institute, Hangzhou, China; ^2^ Shanghai Key Laboratory of Plant Molecular Sciences, College of Life Sciences, Shanghai Normal University, Shanghai, China

**Keywords:** hybrid rice, appearance quality, cooking and taste quality, ecological evolution, chronological evolution

## Abstract

Since the inception of hybrid rice technology 50 years ago, it has not only substantially increased rice yield per unit area but also expedited the development of high-quality rice varieties. However, the evolutionary characteristics of hybrid rice quality remain unclear. To address this gap, it is imperative to leverage more representative and comprehensive hybrid rice resources to analyze phenotypic variation diversity and its primary genetic basis, thereby offering more efficient guidance for molecular breeding. In this study, we selected 2,618 hybrid rice varieties that have been nationally or provincially approved in China over the past 40 years. We analyzed the ecological and chronological evolution characteristics of eight rice quality-related traits: grain length, grain width, grain length-width ratio, chalky grain ratio, chalkiness degree, alkali spreading value, gel consistency, and amylose content (AC). Additionally, we utilized the 'Rice-Navi' system to evaluate the primary molecular basis underlying this evolution. The results revealed that among the eight traits, the coefficient of variation for chalkiness degree was the highest at 0.88, whereas the lowest value of 0.07 was observed for grain width. Significant correlations were found among these traits. The phenotypic evolution results for six major ecological types-Early-season cultivation of indica in South China, Late-season cultivation of indica in South China, Mid-season cultivation of indica in the upper reaches of the Yangtze River, Early-season cultivation of indica in the middle and lower reaches of the Yangtze River, Mid-season cultivation of indica in the middle and lower reaches of the Yangtze River, and Late-season cultivation of indica in the middle and lower reaches of the Yangtze River-indicated that, except for E4, the quality of rice in the other five major ecological types exhibited a significant chronological improvement trend. This trend was highly correlated with the utilization of major superior alleles. Concurrently, the primary genetic background of hybrid rice quality displayed certain ecological diversity characteristics. Overall, this study elucidated the evolutionary characteristics of appearance quality and cooking and taste quality of hybrid rice in southern China from both ecological and chronological perspectives, providing valuable data support for the efficient molecular improvement of rice quality.

## 1 Introduction

Rice is a crucial crop and the staple food for over half of the world’s population (Muthayya et al., 2014). Since Yuan Longping’s invention of hybrid rice production technology in 1973, the widespread utilization of heterosis has significantly enhanced rice yield per unit area, often referred to as the second green revolution in rice (Qian et al., 2016). Despite the successful promotion of hybrid rice, which has substantially increased grain yield, there remains considerable room for improvement in terms of appearance, cooking, and taste qualities compared to conventional rice (Zhao, 2008). As the economy develops and per capita consumption capacity improves, consumer demand for high-quality rice is gradually rising. Consequently, future rice breeders should focus more on cultivating new varieties that combine high quality with high yield (Chen et al., 2023). The appearance quality and cooking and taste quality of rice are the primary indicators for evaluating high-quality rice, directly reflecting its overall quality (Zhang, 2007; Ahmed et al., 2020). Transparency, chalkiness, and grain shape are the main direct indicators for determining appearance quality, while gel consistency, gelatinization temperature, and amylose content are the key parameters for assessing cooking and taste quality.

Appearance quality and cooking and taste quality are both quantitative traits controlled by multiple genes, with numerous genes regulating related traits having been cloned. *GS3* is a major gene that controls grain size and plays a negative regulatory role in regulating grain and organ size (Fan et al., 2006; Mao et al., 2010). *GS3* belongs to one of the gamma subunits of rice Gγ proteins, and recent studies have revealed its simultaneous regulation of heat tolerance and alkali tolerance in rice (Kan et al., 2022; Zhang et al., 2023). *Grain Length on Chromosome 7* (*GL7*) regulates both grain length and width by encoding a homologous protein of *Arabidopsis* LONGIFOLIA, which controls cell longitudinal elongation. The 17.1-kb tandem repeat at the *GL7* locus upregulates *GL7* expression and downregulates neighboring negative regulatory factors, thereby increasing rice grain length and improving appearance quality (Wang et al., 2015). *OsLG3*, as a positive regulator of grain length, can enhance rice yield without compromising quality (Yu et al., 2017). *qLGY3* encodes a transcription factor, *OsMADS1*, containing the *MADS* domain, which is a key downstream effector of G-protein βγ dimers. The alternative splicing of *OsMADS1* ^lgy3^ results in elongated grains, improving both quality and yield (Liu et al., 2018). *GW3p6* (*qLGY3*) significantly boosts rice yield and increases grain weight and length but does not affect other agronomic traits (Wang et al., 2019). *GL3.2* is a homolog of *OsCYP78A13*; loss-of-function mutants of *CYP78A13* exhibit a large embryo phenotype at the expense of the endosperm, without altering overall seed size, indicating that CYP78A13 regulates the embryo/endosperm size balance (Xu et al., 2015a). *GS5* encodes a serine carboxypeptidase that positively regulates rice grain size (Li et al., 2011). Two key single-nucleotide polymorphisms (SNPs) in the *GS5* promoter region lead to differential expression in young rice panicles, determining variations in grain size (Xu et al., 2015b).

*GW5* encodes a 144-amino acid nuclear localization protein containing a nuclear localization signal and an arginine-rich region (Weng et al., 2008). *GW5* regulates cell division through the ubiquitin–proteasome pathway, controlling grain width and weight by altering the number of glume cells (Shomura et al., 2008; Liu et al., 2017; Duan et al., 2017). High expression of the *GW8* (*OsSPL16*) gene promotes cell division and grain filling, positively regulating grain width and yield (Wang et al., 2012). *GW8*, a transcription factor containing the SBP domain, regulates rice grain width and can directly bind to the *GW7* promoter to inhibit its expression (Wang et al., 2015). *Chalk5* encodes a vacuolar membrane proton-transporting pyrophosphatase with non-mechanical pyrophosphate hydrolyzing and proton-transporting activities, affecting chalkiness formation in rice grains (Li et al., 2014). *ALK* encodes soluble starch synthase II, which controls rice gelatinization temperature. Changes in amino acids in the ALK gene product can alter starch synthase activity, affecting the synthesis of medium-length branch chains of amylopectin, changing crystal layer structure, and ultimately influencing gelatinization temperature (Gao et al., 2003). The rice *Wx* gene encodes granule-bound starch synthase (GBSS), the main gene controlling amylose synthesis and directly affecting amylose content in rice endosperm and pollen (Wang et al., 1990; Huang et al., 2020). The synthesis of resistant starch depends on the high expression of the *Waxy* ^a^ (*Wx* ^a^) allele (Zhou et al., 2016). In addition to affecting grain starch content, *Wx* (*qGC-6*) regulates gel consistency (Su et al., 2011).

Since the inception of hybrid rice technology 50 years ago, it has not only significantly increased rice yield per unit area but also accelerated the development of high-quality rice varieties, making substantial contributions to ensuring global food security. However, the evolutionary characteristics and their primary genetic bases of hybrid rice remain unclear. In this study, we utilized hybrid rice varieties that have been nationally or provincially approved in China over the past 40 years to analyze the ecological and chronological evolution characteristics of appearance quality, cooking, and taste quality traits. Additionally, we employed the “RiceNavi” system (Wei et al., 2021) to evaluate the main genetic bases underlying these traits, aiming to provide valuable data support for the efficient molecular breeding of high-quality rice in the future.

## 2 Materials and methods

### 2.1 Source of material and quality trait data

A total of 2,618 experimental materials were sourced from the National Rice Regional Trial Standard Sample Library in Fuyang, Hangzhou, encompassing the main representative hybrid rice varieties approved by national and provincial governments over the past 40 years. The breeding age and ecological classification data for all experimental materials were obtained from the National Crop Variety Testing and Operation Management Platform (http://202.127.45.151/NTP/login.jsp) and the National Rice Data Center (https://www.ricedata.cn/variety/). The phenotype data for rice appearance quality were derived from the phenotypic experiments of hybrid rice (Gu et al., 2023), while the genotype data were obtained from the sequencing results of previous studies and the “RiceNavi” system (Wei et al., 2021).

### 2.2 Ecological and chronological distribution of materials

Based on the ecological characteristics of rice cultivation in China and the latest method for dividing the same adaptive ecological regions, the experimental materials were classified into six ecotypes: early-season cultivation of *indica* in South China (E1), late-season cultivation of *indica* in South China (E2), mid-season cultivation of *indica* in the upper reaches of the Yangtze River (E3), early-season cultivation of *indica* in the middle and lower reaches of the Yangtze River (E4), mid-season cultivation of *indica* in the middle and lower reaches of the Yangtze River (E5), and late-season cultivation of *indica* in the middle and lower reaches of the Yangtze River (E6). These ecotypes cover the most important ecological types in the southern rice region of China (**Supplementary Table S1**). The chronological classification of materials was divided into three stages—before 2000 (Y1), 2001–2010 (Y2), and 2011–2020 (Y3)—with varieties accounting for 13.77%, 48.37%, and 34.16%, respectively (**Supplementary Table S2**).

### 2.3 Software name and purpose

The details can be found in **Supplementary Table S4**.

## 3 Results

### 3.1 AQ and CTQ performance of 2,618 hybrid rice varieties

An investigation was conducted on the quality traits of 2,618 hybrid rice varieties, analyzing eight phenotype data (**Table 1**). The results revealed substantial variation among the quality traits, with coefficients of variation ranging from 6.54% to 87.56%. The coefficients of variation for chalky grain ratio (CR) and chalkiness degree (CS) were the highest, at 64.36% and 87.56%, respectively. The coefficient of variation for grain shape ranged from 6% to 8%. The significant variation observed among various quality traits provides favorable conditions for screening high-quality germplasm resources.

**Table 1:** Statistical analysis of AQ and CTQ traits of 2,618 hybrid rice varieties.

Appearance quality traits	Maximum	Minimum	Mean	Standard error	Standard deviation	Variance	Coefficient of variation
Grain length (GL; mm)	7.37	4.74	6.28	0.01	0.41	0.17	0.07
Grain width (GW; mm)	2.83	1.59	2.11	0.00	0.18	0.03	0.08
Grain length–width ratio (GLWR; mm)	4.17	1.70	3.01	0.01	0.33	0.11	0.11
Chalky grain ratio (CR; %)	100.00	1.53	26.05	0.33	16.77	281.07	0.64
Chalkiness degree (CS; %)	85.89	0.49	7.71	0.13	6.75	45.57	0.88
Alkali spreading value (ASV; grade)	7.00	3.00	5.65	0.02	0.85	0.73	0.15
Gel consistency (GC; mm)	100.00	30.00	69.53	0.37	18.56	344.39	0.27
Amylose content (AC; %)	31.64	2.30	20.45	0.08	3.86	14.90	0.19

AQ, appearance quality; CTQ, cooking and taste quality.

### 3.2 Correlation between AQ and CTQ of 2,618 hybrid rice samples

We conducted a correlation analysis on the phenotype data of related traits, and the results indicated that there was a certain correlation among various traits, with most traits showing extremely significant correlations (**Table 2**). There was a very significant negative correlation between grain length (GL) and grain width (GW), a very significant positive correlation between GL and grain length–width ratio (GLWR), and a very significant negative correlation between GW and GLWR. Additionally, there was a highly significant positive correlation between CR and CS. There was a highly significant negative correlation between chalkiness traits (CR and CS), alkali spreading value (ASV), and gel consistency (GC) and a highly significant positive correlation with amylose content (AC). These findings suggest that there is a significant negative or positive correlation among rice quality traits. Therefore, in the breeding process aimed at improving the quality traits of hybrid rice, it is essential to comprehensively consider various traits to achieve a perfect balance.

**Table 2:** Correlation analysis between AQ and CTQ traits of 2,618 hybrid rice varieties.

Traits	GL (mm)	GW (mm)	GLWR (mm)	CR (%)	CS (%)	ASV (grade)	GC (mm)
GW (mm)	−0.254**						
GLWR (mm)	0.715**	−0.848**					
CR (%)	−0.173**	0.582**	−0.514**				
CS (%)	−0.187**	0.406**	−0.394**	0.879**			
ASV (grade)	−0.078**	0.074**	−0.074**	−0.158**	−0.159**		
GC (mm)	0.170**	−0.174**	0.230**	−0.380**	−0.325**	−0.081**	
AC (%)	−0.046*	0.375**	−0.308**	0.378**	0.242**	0.189**	−0.527**

Asterisk denotes statistically significant differences *p < 0.05; **p < 0.01.

AQ, appearance quality; CTQ, cooking and taste quality; GL, grain length; GW, grain width; GLWR, grain length–width ratio; CR, chalky grain ratio; CS, chalkiness degree; ASV, alkali spreading value; GC, gel consistency; AC, amylose content.

### 3.3 The evolution trend of AQ and CTQ among different eco-region over time

**Figure 1:**
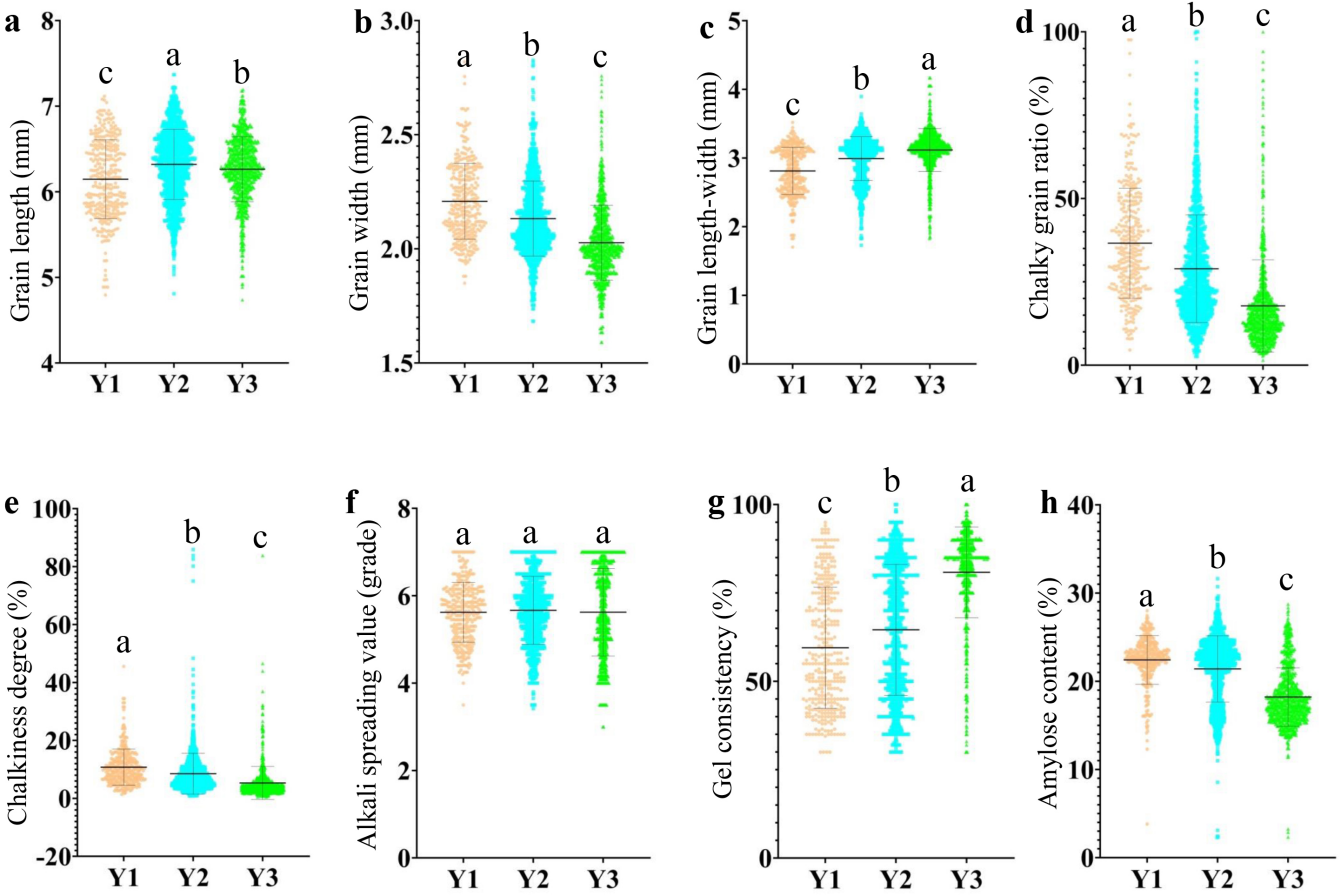
The evolution of AQ and CTQ traits in different eras. Y1 represents varieties before 2000, Y2 represents varieties from 2001 to 2010, and Y3 represents varieties from 2011 to 2020 (**Supplementary Table S2**). The letters a, b, and c indicate significant differences in least significant difference *p* < 0.05. **(A)** The evolution of GL in different eras. **(B)** The evolution of GW in different eras. **(C)** The evolution of GLWR in different eras. **(D)** The evolution of CR in different eras. **(E)** The evolution of CS in different eras. **(F)** The evolution of ASV in different eras. **(G)** The evolution of GC in different eras. **(H)** The evolution of AC in different eras. AQ, appearance quality; CTQ, cooking and taste quality; GL, grain length; GW, grain width; GLWR, grain length–width ratio; CR, chalky grain ratio; CS, chalkiness degree; ASV, alkali spreading value; GC, gel consistency; AC, amylose content.

The quality traits exhibited different trends over time, specifically manifesting as increased grain length, decreased grain width, a significant reduction in chalkiness, and a gradual decrease in amylose content. The trend of alkali spreading value changed with the evolution of time, with relatively minor fluctuations (**Figure 1**). This result indicates that the high-quality rate of hybrid rice has been gradually increasing in China.

**Figure 2:**
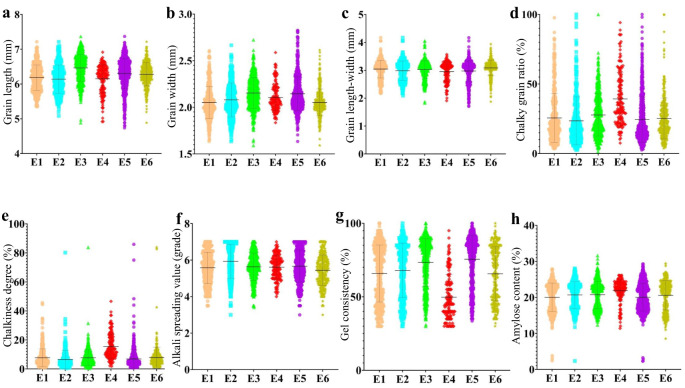
The evolution of AQ and CTQ traits in different ecotypes. E1, E2, E3, E4, E5, and E6 represent different ecotypes (**Supplementary Table S1**). **(A)** The evolution of GL in different ecotypes. **(B)** The evolution of GW in different ecotypes. **(C)** The evolution of GLWR in different ecotypes. **(D)** The evolution of CR in different ecotypes. **(E)** The evolution of CS in different ecotypes. **(F)** The evolution of ASV in different ecotypes. **(G)** The evolution of GC in different ecotypes. **(H)** The evolution of AC in different ecotypes. AQ, appearance quality; CTQ, cooking and taste quality; GL, grain length; GW, grain width; GLWR, grain length–width ratio; CR, chalky grain ratio; CS, chalkiness degree; ASV, alkali spreading value; GC, gel consistency; AC, amylose content.

This study classified 2,618 materials into six ecotypes (E1, E2, E3, E4, E5, and E6) based on different planting areas and maturity periods (**Supplementary Table S1**). In different ecotypes, quality traits exhibited varying degrees of variation. The results indicated that the variation amplitude of appearance quality (AQ) and cooking and taste quality (CTQ) traits differed across ecotypes (**Figure 2**). The quality of hybrid rice varieties in ecotypes E2 and E6 was relatively high. In contrast, the quality of hybrid rice varieties in ecotype E4 was relatively poor, particularly in terms of chalkiness and gel consistency.

**Figure 3:**
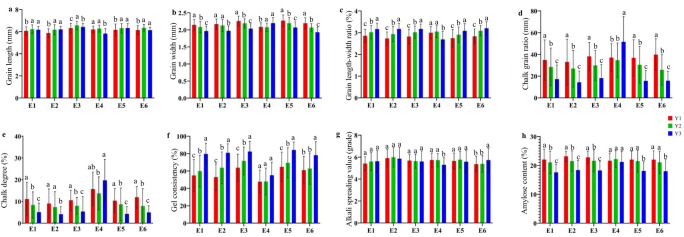
The evolution of AQ and CTQ over time in six ecotypes. E1, E2, E3, E4, E5, and E6 represent different ecotypes (**Supplementary Table S1**). Y1 represents varieties before 2000, Y2 represents varieties from 2001 to 2010, and Y3 represents varieties from 2011 to 2020 (**Supplementary Table S2**). The letters a, b, and c indicate significant differences in least significant difference (*p* < 0.05). **(A)** The evolution of GL in different eras. **(B)** The evolution of GW over time in different ecotypes. **(C)** The evolution of GLWR over time in different ecotypes. **(D)** The evolution of CR over time in different ecotypes. **(E)** The evolution of CS over time in different ecotypes. **(F)** The evolution of ASV over time in different ecotypes. **(G)** The evolution of GC over time in different ecotypes. **(H)** The evolution of AC over time in different ecotypes. AQ, appearance quality; CTQ, cooking and taste quality; GL, grain length; GW, grain width; GLWR, grain length–width ratio; CR, chalky grain ratio; CS, chalkiness degree; ASV, alkali spreading value; GC, gel consistency; AC, amylose content.

Phenotypic analysis was conducted on the six different ecotypes according to their chronological changes. The results indicate that with the change of age, there is also a certain range of changes in AQ and CTQ traits (**Figure 3**). Grain shape exhibited a small range of changes, with variation differences of 1–2 mm. There were significant differences in the variation of chalkiness traits, with CR ranging from 15% to 55% and CS ranging from 2.5% to 22.5%. The range of changes in other traits varied, with the smallest being the range of changes in ASV, with a difference of less than 1%. The largest is the change in AC, with a range of changes of approximately 10%. Additionally, with the passage of time, the AQ and CTQ traits showed a favorable trend of change. Except for E4, in the other five ecotypes, the same quality trait exhibited similar changes, with GL becoming longer and GW becoming shorter. The CR and CS gradually decreased, and the ASV became higher. However, GC and AC showed consistent changes in each ecological type, with higher GC and lower AC.

### 3.4 Analysis of the impact of AQ and CTQ genes on related phenotypes

Based on the genome resequencing of 2,618 hybrid rice varieties from previous studies (Gu et al., 2023), we utilized the “RiceNavi” system (Wei et al., 2021) to perform genotype analysis on genes related to quality traits (**Supplementary Table S3**). We organized the genotype and phenotype data and conducted a correlation analysis of *R* ^2^ (**Table 3**). The results showed that *GS3* has the highest correlation with GL, GW, and GLWR. The *Waxy* gene has the highest correlation with CR, CS, GC, and AC. *ALK* has the highest correlation with ASV.

**Table 3:** *R* ^2^ between 16 genes and eight traits.

Genes	Altered allele function	GL	GW	GLWR	CR	CS	ASV	GC	AC
*GS3*	Increasing grain length and width	0.31662	0.30318	0.50189	0.18863	0.11042	0.00151	0.08692	0.09508
*GL3.2/CYP78A5*	Decreasing grain length	0.03768	0.07007	0.00687	0.05745	0.02009	0.01640	0.00001	0.00475
*GL3.3/qTGW3*	Increasing grain length	/	/	/	/	/	/	/	/
*OsLG3*	Increasing grain length	0.00597	0.00784	0.01100	0.03672	0.02175	0.00402	0.04521	0.03986
*qLGY3/OsMADS1/GW3p6/OsLG3b*	Increasing grain length	0.01741	0.00089	0.00201	0.00106	0.00115	0.00966	0.00604	0.00001
*GS5*(*GS5-2*)	Decreasing grain width	0.01954	0.05337	0.06715	0.07309	0.04243	0.00924	0.06450	0.07828
*GS5*(*GS5-1*)	Decreasing grain width	0.02697	0.06936	0.08526	0.08168	0.05244	0.01072	0.06398	0.07140
*GW5/GSE5/qSW5*	Increasing grain width	0.00771	0.14323	0.09798	0.10546	0.05785	0.00242	0.00312	0.00976
*GS6/DLT/OsGRAS-32/SMOS2/D62*	Decreasing grain width	0.03131	0.00002	0.00810	0.01907	0.01322	0.01000	0.04963	0.07904
*GW7*	Increasing grain length	0.00020	0.00073	0.00385	0.00042	0.00033	0.02024	0.00375	0.00033
*GE/CYP78A13/BG2*(1)	Increasing grain length and grain width	0.00000	0.00384	0.00478	0.00583	0.00722	0.03672	0.02982	0.01626
*GE/CYP78A13/BG2*(2)	Increasing grain length and grain width	0.02425	0.01450	0.00010	0.00342	0.00679	0.00902	0.01026	0.00094
*OsSNB*	Increasing grain length	0.00056	0.00034	0.00081	0.00024	0.00021	0.00080	0.00019	0.00051
*OsRAE2*	Increasing grain length	0.00018	0.00016	0.00024	0.00078	0.00207	0.00018	0.00064	0.00003
*GW8* (*gw8* ^Basmati^)	Increasing grain width	0.00507	0.01167	0.01866	0.00193	0.00040	0.00001	0.00452	0.00329
*GW8* (*GW8*-5174C>A)	Increasing grain width	0.05999	0.02838	0.00027	0.01974	0.00762	0.00800	0.00740	0.03225
*Chalk5*	Decreasing chalkiness	0.02883	0.07858	0.09458	0.09071	0.05565	0.00907	0.06673	0.08576
*Waxy* (*Wx^lv^/Wx^a^/Wx^in^/Wx^op^/Wx^hp^*)	Increasing amylose content	0.03368	0.17400	0.17213	0.25605	0.17095	0.00105	0.30360	0.53234
*Waxy* (*Wx^mp^*)	Decreasing amylose content	/	/	/	/	/	/	/	/
*Waxy* (*Wx^op/hp^*)	Decreasing amylose content	/	/	/	/	/	/	/	/
*Waxy* (*Wx^in^*/*Wx^mw^*)	Increasing amylose content	0.00110	0.00047	0.00107	0.00157	0.00152	0.00008	0.00007	0.00102
*Waxy* (*Wx^a^*)	Increasing amylose content	0.03356	0.19589	0.18763	0.25124	0.13678	0.00124	0.35888	0.50622
*ALK* (*ALK*-3797A>G)	Increasing gelatinization temperature	/	/	/	/	/	/	/	/
*ALK* (*ALK*-4328C>T)	Decreasing gelatinization temperature	0.00193	0.06458	0.05285	0.14447	0.10229	0.35383	0.05532	0.09852

Numbers represent correlation coefficient (*R* ^2^). “/” indicates that there is only one allele form in the hybrid rice varieties studied. (1), (2), (3), (4), (5), and (6) represent different alleles.

GL, grain length; GW, grain width; GLWR, grain length–width ratio; CR, chalky grain ratio; CS, chalkiness degree; ASV, alkali spreading value; GC, gel consistency; AC, amylose content.

**Figure 4:**
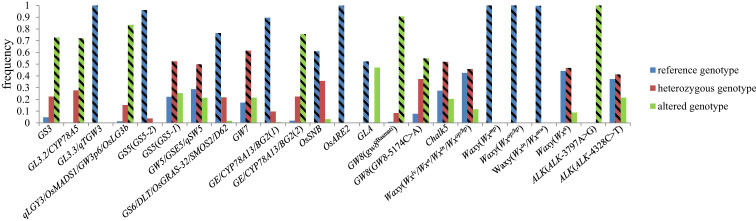
The different genotype frequencies of 16 genes in 2,618 hybrid rice varieties. The rectangle with diagonal lines represents the genotype with higher frequency utilization in hybrid rice.

We further analyzed the allele forms of 16 genes in hybrid rice and found that most of the genes affecting AQ and CTQ in breeding were applied in homozygous genotypes, while a small number were applied in heterozygous genotypes, such as *GS5*, *GS6*, *Chalk5*, *Waxy* (*Wx^lv^/Wx^a^/Wx^in^/Wx^op^/Wx^hp^*), *Waxy* (*Wx^a^*), and *ALK*(*ALK-4328C>T*) (**Figure 4**). The results have a certain guiding significance for the genetic improvement of maintainer and restorer lines in future hybrid rice breeding.

### 3.5 The evolution of excellent genes with age type

**Figure 5:**
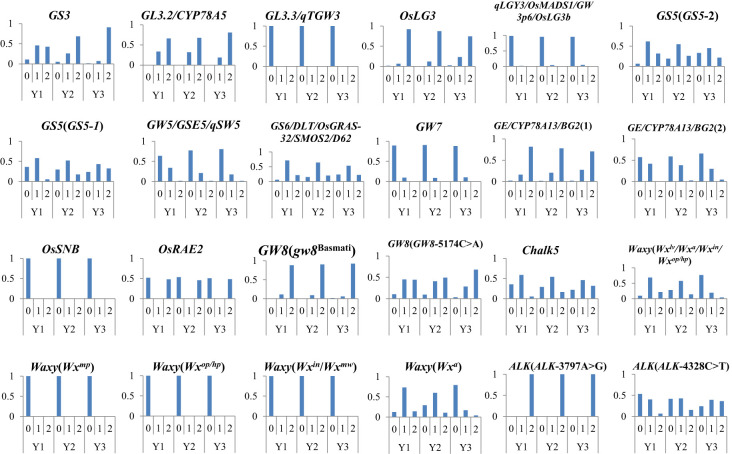
The variation allele frequency of genes affecting AQ and CTQ across different eras. The vertical axis represents frequency. Y1 represents varieties before 2000, Y2 represents varieties from 2001 to 2010, and Y3 represents varieties from 2011 to 2020 (**Supplementary Table S3**). “0” represents homozygous genotype (reference genotype), “1” represents heterozygous genotype, and “2” represents alterant genotype. AQ, appearance quality; CTQ, cooking and taste quality.

We have integrated representative approved varieties from the past 40 years, sequenced each sample to obtain genotype data, and screened out excellent genes that significantly impact AQ and CTQ. We analyzed the allele frequency of these excellent genes. The results showed that with the change of year interval, the allele frequency of excellent genes exhibited a hierarchical change (**Figure 5**). Among these, the frequency of the altered genotype in *GS3*, *GS5* (*GS5-1*), *GW8* (*GW8*-5174C>A), *Chalk5*, and *ALK* (*ALK*-4328C>T) has been increasing year by year. The frequency of the reference genotype in *GW5* has been decreasing year by year. The frequency of the alterant genotype in *Waxy* (*Wx^lv^/Wx^a^/Wx^in^/Wx^op^/Wx^hp^*) and *Waxy* (*Wx* ^a^) has been decreasing year by year.

In hybrid rice breeding, combinations of reference alleles of *GL3.3*, *GW3p6*, *GW5*, *GW7*, *BG2(2)*, *OsSN*B, and *OsARE2*; alterant alleles of *GS3*, *GL3.2*, *OsLG3*, *BG(1)*, and *GW8*; and heterozygous alleles of *GS5*, *GS6*, and *Chalk5* may result in better AQ. The combination of the reference alleles of *Waxy* (*Wx^mp^*), *Waxy* (*Wx^op/hp^*), and *Waxy* (*Wx^in^*/*Wx^mw^*); the alterant allele of *ALK*(*AIK*-3797A>G); and the heterozygous alleles of *Waxy* (*Wx^lv^/Wx^a^/Wx^in^/Wx^op^/Wx^hp^*), *Waxy* (*Wx^a^*), and *ALK*(*AIK*-4328C>T) may result in better CTQ.

### 3.6 The evolution of excellent genes with ecotypes

**Figure 6:**
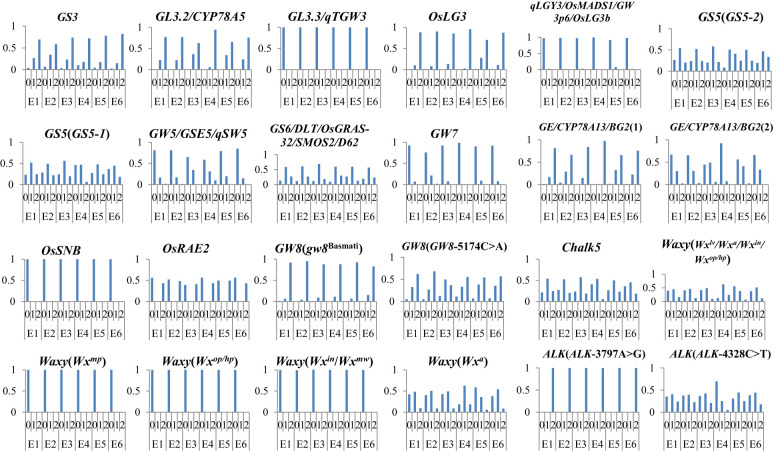
The variation allele frequency of genes affecting AQ and CTQ in different ecotypes. The vertical axis represents frequency. E1, E2, E3, E4, E5, and E6 represent different ecotypes (**Supplementary Table S1**). “0” represents homozygous genotype (reference genotype), “1” represents heterozygous genotype, and “2” represents alterant genotype. AQ, appearance quality; CTQ, cooking and taste quality.

We further investigated the allele frequency of variations in 16 genes affecting AQ and CTQ across different ecotypes (**Figure 6**). We found that grain length showed a slight increase from E1 to E6, with the largest change observed in E3 (**Figure 3**). According to the variation of allele frequencies of excellent genes, the allele frequency of the *GL3.2* heterozygous genotype was the lowest in E3. Grain width reached its highest in E3 and its lowest in E1 (**Figure 2**). From the variation of allele frequencies of excellent genes, the *GS5* heterozygous genotype had the lowest variation allele frequency in E3, while the *GW5* reference genotype and *GW7* reference genotype had relatively low allele frequencies in E1.

Chalkiness exhibited different changes across the six different ecotypes, with CR and CS being the highest in E4 and lower in E1 and E2 (**Figure 2**). The allele frequencies of *Chalk5*, a major gene affecting chalkiness, varied among the six ecotypes. The *Chalk5* heterozygous genotype had higher allele frequencies in E1, E2, and E3. The allele frequencies of the *Chalk5* reference genotype were higher in E4. AC differed across different ecotypes. In E1, E2, E3, E5, and E6, AC showed a decreasing trend. E4 consistently exhibited high AC (**Figure 2**). By analyzing the variation allele frequency of *Waxy*, the allele frequencies of *Waxy* (*Wx^lv^/Wx^a^/Wx^in^/Wx^op^/Wx^hp^*) and *Waxy* (*Wx* ^a^) heterozygous genotypes were the highest in E4.

Among the six different ecological types, ASV was the highest in E2, but the ALK reference genotype had the highest allele frequency in E4. The combined alleles of ecotypes with better AQ tended to exhibit *GL3.3*, *GW3p*6, *GW5*, *GW7*, and *OsSNB* as reference alleles and *GS3*, *GL3.2*, *OsLG*3, *BG2(1)*, *OsARE2*, and *GW8* (*gw8* ^Basmati^) as alterant alleles, with *GS5*, *GS6*, *GW8*(*GW8*-5174C>A), and *Chalk5* as heterozygous alleles. The combined alleles of ecotypes with better CTQ tended to exhibit *Waxy* (*Wx^mp^*), *Waxy* (*Wx^op/hp^*), and *Waxy* (*Wx^in^*/*Wx^mw^*) as reference alleles; *ALK*(*ALK*-3797A>G) as an alterant allele; and *Waxy* (*Wx^lv^/Wx^a^/Wx^in^/Wx^op^/Wx^hp^*), *Waxy* (*Wx* ^a^), and *ALK* (*ALK*-4328C>T) as heterozygous alleles. These results indicate a certain correlation between genes and quality, but quality is determined by comprehensive factors, including environmental factors.

## 4 Discussion

### 4.1 Characteristics of AQ and CTQ traits of hybrid rice

Grain shape and chalkiness are the main traits that determine AQ. Grain shape-related traits include grain length, grain width, grain thickness, and length–width ratio, while chalkiness can be categorized into white core, white belly, and white back based on location. CTQ refers to the comprehensive evaluation of sensory indicators such as smell, color, shape, palatability, taste, and other sensory indicators after cooking under specific conditions. Current evidence suggests that the main indicators affecting CTQ are amylose content, protein content, fat content, gel consistency, alkali spreading value (gelatinization temperature), and cooking method (Zhou et al., 2019).

We analyzed the correlation coefficients of eight traits, including GL, GW, GLWR, and CR, among 2,837 hybrid rice varieties (**Table 2**). This study demonstrated that the appearance quality traits of rice not only acted independently but also influenced each other. It was found that AC was significantly correlated with CR, CS, and GC. CR was significantly correlated with CS (0.879**). GLWR was significantly correlated with GC, CS, and CR. These results are consistent with those of previous studies (Li et al., 1995; Xu et al., 2004; Lei et al., 2010; Yang et al., 2023). Therefore, elongated rice grains generally exhibit low chalkiness. Reducing grain width, increasing grain length, and grain length–width ratio can reduce chalkiness and improve the appearance quality of rice. High chalkiness is the most prominent problem in the quality of hybrid rice and is also the main target of hybrid rice breeding for high quality. Thus, it is particularly important to select parents with low chalkiness. In terms of quality traits of hybrid rice, the selection of hybrid parents whose main goal is to improve the AQ and CTQ of hybrid rice should be based on the quality level of parents and the genetic diversity of germplasm.

### 4.2 Evolution trend of AQ and CTQ

In general, for high-quality rice, it is required to have longer GL, smaller GW, low CR, low CS, higher ASV, higher GC, and lower AC (Li et al., 1987; Tang, 1987; Fan et al., 2005; Wang et al., 2007). In this study, we found that the GL of hybrid rice gradually increases, GW gradually decreases, AC gradually decreases, and chalkiness gradually improves with the passage of time in China. Fang found that the number of high-quality hybrid rice varieties has been increasing year by year in China (Fang et al., 2020), and the result of this study also reflects that the quality rate of hybrid rice varieties in our country has been increasing year by year. This may be due to the improved quality of some hybrid rice parents (Feng et al., 2017).

In terms of ecotypes, the quality of rice in South China was better than that in the middle and lower reaches of the Yangtze River. In particular, the quality of E4 has not been significantly improved in recent decades, but chalkiness and ASV were even worse. There may be two main reasons for the poor quality of E4 rice. For one thing, early rice has a short growth period, and in order to increase yield, the selection process often tends to favor wide grains. The synergistic effect of chalkiness in wide-grain genes leads to an increase in chalkiness and a decrease in quality. In contrast, around mid-June, the E4 grain filling coincides with the continuous rise in temperature in the middle and lower reaches of the Yangtze River. High temperatures can lead to poor early rice enrichment, reduced polished rice, and increased chalkiness. In the future, breeders may need to develop rice varieties that can produce high-quality rice at high temperatures.

### 4.3 The evolution of some favorable alleles in hybrid rice breeding

In this study, we analyzed the genotypes of 14 genes (*GS3*, *GL3.2/CYP78A5*, *GL3.3/qTGW3*, *OsLG3*, *qLGY3/OsMADS1/GW3p6/OsLG3b*, *GS5*, *GW5/GSE5/qSW5*, *GS6/DLT/OsGRAS-32/SMOS2/D62*, *GW7*, *GE/CYP78A13/BG2*, *OsSNB*, *OsRAE2*, *GW8/OsSPL16*, and *Chalk5*) regulating AQ and two genes (*Waxy* and *ALK*) regulating CTQ in 2,618 hybrid rice varieties. By studying the allele frequency of these genes at different ages, we found that breeders were intentionally or unintentionally utilizing these alleles that are beneficial for improving AQ and CTQ in hybrid rice, leading to the breeding of high-quality hybrid rice varieties. The more notable ones include *GS3*, which regulates GL and GW; *Chalk5*, which regulates chalkiness; and Waxy, which regulates AC. This directly indicates from a molecular perspective that the process of high-quality hybrid rice in China was directly related to these alleles. However, in terms of different ecotypes, some favorable allele utilization still needs to be improved, particularly in E4. In the future, using molecular breeding to pyramid excellent quality genes to improve early rice varieties may be a fast and effective method.

## Data Availability

The data used to support the findings of this study can be made available by the corresponding author upon request.

## References

[B1] Ahmed F. Abro T. F. Kabir M. S. Latif M. A. (2020). “Rice quality: biochemical composition, eating quality, and cooking quality,” in Eds. Costa De Oliveira A. Pegoraro C. Ebeling Viana V. The Future of Rice Demand: Quality Beyond Productivity, eds. Costa De Oliveira A. Pegoraro C. Ebeling Viana V. (Cham: Spirnger), 3–24. doi: 10.1007/978-3-030-37510-2_1

[B2] Chen H. Hu S. K. Tang S. Q. Hu P. S. (2023). Current status and prospect of genetic improvement of rice grain quality. J. Yangtze Univ. (Natural Sience Eitionc d) 20, 110–123. doi: 10.16772/j.cnki.1673-1409.2023.05.002

[B3] Duan P. Xu J. Zeng D. Zhang B. Geng M. Zhang G.. (2017). Natural variation in the promoter of *GSE5* contributes to grain size diversity in rice. Mol. Plant 10, 685–694. doi: 10.1016/j.molp.2017.03.009 28366824

[B4] Fan C. Xing Y. Mao H. Lu T. Han B. Xu C.. (2006). *GS3*, a major QTL for grain length and weight and minor QTL for grain width and thickness in rice, encodes a putative transmembrane protein. Theor. Appl. Genet. 112, 1164–1171. doi: 10.1007/s00122-006-0218-1 16453132

[B5] Fan C. C. Yu X. Q. Xing Y. Z. Xu C. G. Luo L. J. Zhang Q. (2005). The main effects, epistatic effects and environmental interactions of QTLs on the cooking and eating quality of rice in a doubled-haploid line population. Theor. Appl. Genet. 110, 1445–1452. doi: 10.1007/s00122-005-1975-y 15841361

[B6] Fang Y. Zhang W. Chen Y. Hou F. Xu L. Tang C.. (2020). State quo of utilization of high-quality hybrid rice varieties in China during 2001−2017. Acta Agriculturae. Zhejiangensis. 1, 1–14. doi: 10.3969/j.issn.1004-1524.2020.01.01

[B7] Feng F. Li Y. Qin X. Liao Y. Siddique K. H. M. (2017). Changes in rice grain quality of *indica* and *japonica* type varieties released in China from 2000 to 2014. Front. Plant Sci. 8, 1863. doi: 10.3389/fpls.2017.01863 29163589 PMC5671604

[B8] Gao Z. Zeng D. Cui X. Zhou Y. Yan M. Huang D.. (2003). Map-based cloning of the *ALK* gene, which controls the gelatinization temperature of rice. Sci. China C. Life Sci. 46, 661–668. doi: 10.1360/03yc0099 18758723

[B9] Gu Z. Gong J. Zhu Z. Li Z. Feng Q. Wang C.. (2023). Structure and function of rice hybrid genomes reveal genetic basis and optimal performance of heterosis. Nat. Genet. 55, 1745–1756. doi: 10.1038/s41588-023-01495-8 37679493 PMC10562254

[B10] Huang L. Sreenivasulu N. Liu Q. (2020). *Waxy* editing: old meets new. Trends. Plant Sci. 25, 963–966. doi: 10.1016/j.tplants.2020.07.009 32828690

[B11] Kan Y. Mu X. R. Zhang H. Gao J. Shan J. X. Ye W. W.. (2022). TT2 controls rice thermotolerance through SCT1-dependent alteration of wax biosynthesis. Nat. Plants 8, 53–67. doi: 10.1038/s41477-021-01039-0 34992240

[B12] Lei D. Xie F. Chen L. (2010). Genetic and correlation analysis of grain appearance quality in hybrid rice. Res. Agric. Modernization 2, 212–215. doi: CNKI:SUN:XNYX.0.1996-S1-000

[B13] Li Y. Fan C. Xing Y. Jiang Y. Luo L. Sun L.. (2011). Natural variation in *GS5* plays an important role in regulating grain size and yield in rice. Nat. Genet. 43, 1266–1269. doi: 10.1038/ng.977 22019783

[B14] Li Y. Fan C. Xing Y. Yun P. Luo L. Yan B.. (2014). *Chalk5* encodes a vacuolar H(+)-translocating pyrophosphatase influencing grain chalkiness in rice. Nat. Genet. 46, 398–404. doi: 10.1038/ng.2923 24633159

[B15] Li S. Li H. Zhou K. Ma Y. (1995). Genetic analysis of exterior quality traits in hybrid rice. Southwest China J. Agric. Sci. 3, 197–201. doi: CNKI:SUN:XNYX.0.1996-S1-000

[B16] Li X. Pan X. Gu M. (1987). A study on grain quality of rice for commercial varieties. Jiangsu J. Agr. Sci. 1, 1–8. doi: 10.16872/j.cnki.1671-4652.1987.01.001

[B17] Liu J. Chen J. Zheng X. Wu F. Lin Q. Heng Y.. (2017). *GW5* acts in the brassinosteroid signalling pathway to regulate grain width and weight in rice. Nat. Plants 3, 17043. doi: 10.1038/nplants.2017.43 28394310

[B18] Liu Q. Han R. Wu K. Zhang J. Ye Y. Wang S.. (2018). G-protein βγ subunits determine grain size through interaction with MADS-domain transcription factors in rice. Nat. Commun. 9, 852. doi: 10.1038/s41467-018-03047-9 29487282 PMC5829230

[B19] Mao H. Sun S. Yao J. Wang C. Yu S. Xu C.. (2010). Linking differential domain functions of the GS3 protein to natural variation of grain size in rice. Proc. Natl. Acad. Sci. U. S. A. 107, 19579–19584. doi: 10.1073/pnas.1014419107 20974950 PMC2984220

[B20] Muthayya S. Sugimoto J. D. Montgomery S. Maberly G. F. (2014). An overview of global rice production, supply, trade, and consumption. Ann. New York Acad. Sci. 1324, 7–14. doi: 10.1111/nyas.2014.1324.issue-1 25224455

[B21] Qian Q. Guo L. Smith S. M. L. Li J. (2016). Breeding high-yield superior quality hybrid super rice by rational design. Natl. Sci. Rev. 3, 283–294. doi: 10.1093/nsr/nww006

[B22] Shomura A. Izawa T. Ebana K. Ebitani T. Kanegae H. Konishi S.. (2008). Deletion in a gene associated with grain size increased yields during rice domestication. Nat. Genet. 40, 1023–1028. doi: 10.1038/ng.169 18604208

[B23] Su Y. Rao Y. Hu S. Yang Y. Gao Z. Zhang G.. (2011). Map-based cloning proves *qGC-6*, a major QTL for gel consistency of *japonica*/*indica* cross, responds by *Waxy* in rice (Oryza sativa L.). Theor. Appl. Genet. 123, 859–867. doi: 10.1007/s00122-011-1632-6 21698394

[B24] Tang S. (1987). A Study on the cooking and eating qualities of chinese hybrid rices (*O. Sativa* L.). Scientia Agricultura Sin. 5, 17–22. doi: 10.3864/j.issn.0578-1752.1987-20-05-17-22

[B25] Wang S. Li S. Liu Q. Wu K. Zhang J. Wang S.. (2015). The *OsSPL16-GW7* regulatory module determines grain shape and simultaneously improves rice yield and grain quality. Nat. Genet. 47, 949–954. doi: 10.1038/ng.3352 26147620

[B26] Wang L. Q. Liu W. J. Xu Y. He Y. Q. Luo L. J. Xing Y. Z.. (2007). Genetic basis of 17 traits and viscosity parameters characterizing the eating and cooking quality of rice grain. Theor. Appl. Genet. 115, 463–476. doi: 10.1007/s00122-007-0580-7 17593343

[B27] Wang C. Tang S. Zhan Q. Hou Q. Zhao Y. Zhao Q.. (2019). Dissecting a heterotic gene through GradedPool-Seq mapping informs a rice-improvement strategy. Nat. Commun. 10, 2982. doi: 10.1038/s41467-019-11017-y 31278256 PMC6611799

[B28] Wang Z. Y. Wu Z. L. Xing Y. Y. Zheng F. G. Guo X. L. Zhang W. G.. (1990). Nucleotide sequence of rice *Waxy* gene. Nucleic. Acids Res. 18, 5898. doi: 10.1093/nar/18.19.5898 2216792 PMC332347

[B29] Wang S. Wu K. Yuan Q. Liu X. Liu Z. Lin X.. (2012). Control of grain size, shape and quality by *OsSPL16* in rice. Nat. Genet. 44, 950–954. doi: 10.1038/ng.2327 22729225

[B30] Wei X. Qiu J. Yong K. Fan J. Zhang Q. Hua H.. (2021). A quantitative genomics map of rice provides genetic insights and guides breeding. Nat. Genet. 53, 243–253. doi: 10.1038/s41588-020-00769-9 33526925

[B31] Weng J. Gu S. Wan X. Gao H. Guo T. Su N.. (2008). Isolation and initial characterization of *GW5*, a major QTL associated with rice grain width and weight. Cell. Res. 18, 1199–1209. doi: 10.1038/cr.2008.307 19015668

[B32] Xu Z. Chen W. Ma D. Lv Y. Zhou S. Liu L. (2004). Correlations between rice grain shapes and main qualitative characteristics. Acta Agronomica Sinica. 9, 894–900. doi: 10.3321/j.issn:0496-3490.2004.09.009

[B33] Xu F. Fang J. Ou S. Gao S. Zhang F. Du L.. (2015a). Variations in *CYP78A13* coding region influence grain size and yield in rice. Plant Cell. Environ. 38, 800–811. doi: 10.1111/pce.2015.38.issue-4 25255828

[B34] Xu C. Liu Y. Li Y. Xu X. Xu C. Li X.. (2015b). Differential expression of *GS5* regulates grain size in rice. J. Exp. Bot. 66, 2611–2623. doi: 10.1093/jxb/erv058 25711711 PMC4986870

[B35] Yang X. Pan Y. Xia X. Qing D. Chen W. Nong B.. (2023). Molecular basis of genetic improvement for key rice quality traits in southern China. Genomics 115, 110745. doi: 10.1016/j.ygeno.2023.110745 37977332

[B36] Yu J. Xiong H. Zhu X. Zhang H. Li H. Miao J.. (2017). *OsLG3* contributing to rice grain length and yield was mined by Ho-LAMap. BMC. Biol. 15, 28. doi: 10.1186/s12915-017-0365-7 28385155 PMC5383996

[B37] Zhang Q. (2007). Strategies for developing green super rice. Proc. Natl. Acad. Sci. U.S.A. 104, 16402–16409. doi: 10.1073/pnas.0708013104 17923667 PMC2034246

[B38] Zhang H. Yu F. Xie P. Sun S. Qiao X. Tang S.. (2023). A Gγ protein regulates alkaline sensitivity in crops. Science 379, eade8416. doi: 10.1126/science.ade8416 36952416

[B39] Zhao J. (2008). Comparsion of grain in yield and quality between hybrid rice and inbred rice in China. Hybrid Rice 23, 1–4. doi: 10.16267/j.cnki.1005-3956.2008.02.005

[B40] Zhou H. Wang L. Liu G. Meng X. Jing Y. Shu X.. (2016). Critical roles of soluble starch synthase *SSIIIa* and granule-bound starch synthase *Waxy* in synthesizing resistant starch in rice. Proc. Natl. Acad. Sci. U.S.A. 113, 12844–12849. doi: 10.1073/pnas.1615104113 27791174 PMC5111662

[B41] Zhou H. Xia D. He Y. (2019). Rice grain quality—traditional traits for high quality rice and health-plus substances. Mol. Breed. 40, 1.

